# Innovative Technique of Vascular Repair in Intra-Operative IVC Rupture During Lumbar Microdiscectomy: A Case Report

**DOI:** 10.5812/atr.11005

**Published:** 2013-12-01

**Authors:** Sandeep Singh, Arun Bhanot, Nipun Bajaj, Pooja Rustagi

**Affiliations:** 1 Primus Super Specialty Hospital, Chankayapuri, New Delhi, India

**Keywords:** Microdiscectomy Complications, Vascular Injury, Inferior Vena Cava Rupture, Anastomosis, Vascular Bypass

## Abstract

**Background:**

Major vascular injury during a spinal surgery is a rare but most dreaded complication.

**Case Presentation:**

A 39 years old female undergoing microscopic lumbar discectomy suddenly developed severe hypotension on table. The procedure was abandoned and the patient turned supine. It was diagnosed to be a major vessel tear and the patient was taken up for immediate successful vascular repair. To best of our knowledge such a repair procedure has not been described in literature.

**Conclusions:**

Majority of such vascular injuries are dealt with primary repair of the defect by a vascular surgeon; however in our case the rent was big and placed on the undersurface making it very difficult for the vascular surgeon to approach or repair it primarily.

## 1. Background

Major vascular injury during a spinal surgery is a rare but most dreaded complication. The reported incidence of such injury during a lumbar disc surgery is 0.01 - 0.05% (1). However many of the cases go unreported. The mortality rate may be as high as 100% (2). Such high mortality rates may be attributed to delay in identifying the injury leading to excessive blood loss beyond control. No wonder iatrogenic major vascular injuries are a nightmare for any spine surgeon. A high index of suspicion, prompt diagnosis and timely appropriate intervention can reduce the mortality rate significantly.

## 2. Case Presentation

**Figure 1.**
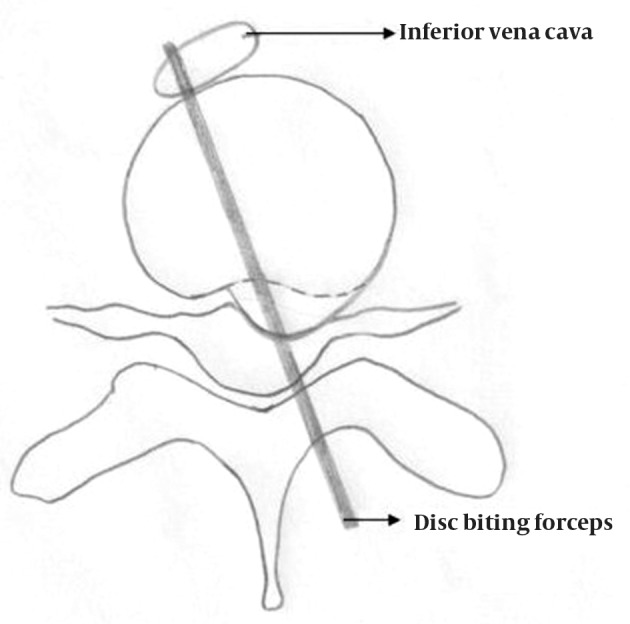
Cross-sectional Diagrammatic Representation of Mechanism of Iatrogenic Injury to the Inferior Vena Cava During Microdiscectomy at L4-L5

**Figure 2.**
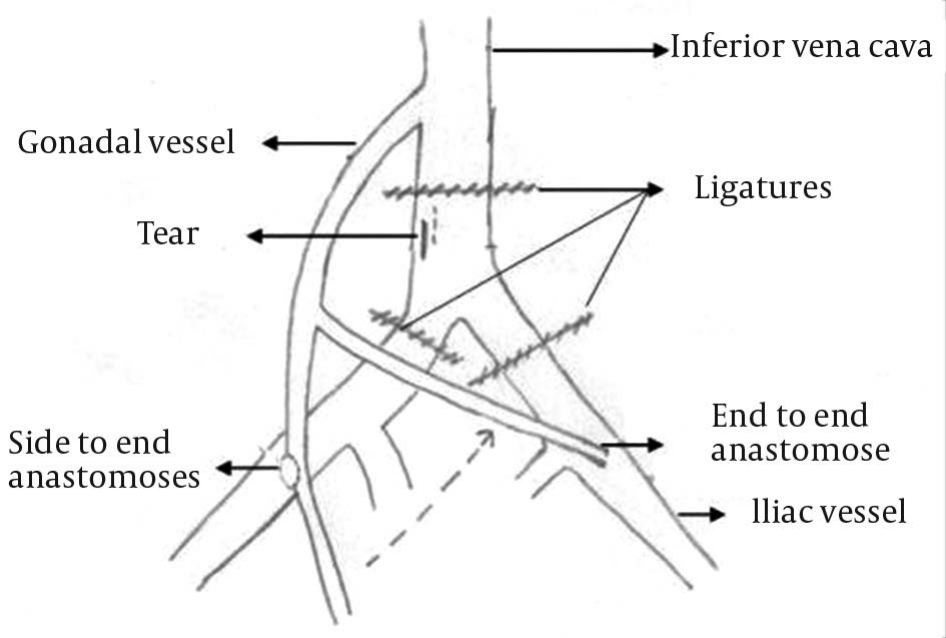
Diagram Showing the Rent in the Inferior Vena Cava and the Vascular Repair Thus Performed

A 39 years old female patient was admitted with a diagnosis of prolapsed intervertebral disc L4-L5 with radiculopathy left lower limb. The patient was taken up for routine microdiscectomy after pre anesthetic checkup. After doing the laminotomy, while the discectomy was being carried out with help of disc biting forceps anesthetists noticed a sudden drop in blood pressure of the patient. Even after checking the wires and cuff when the readings did not improved, it was decided to pack the wound and turn the patient supine. As there was no excessive pulsatile bleeding into the surgical field, major blood vessel injury was not contemplated. The surgical and the anesthetic team assumed it to be a severe drug allergic reaction. However an urgent ultrasound of abdomen was called for, which revealed frank collection in the abdomen. Blood was aspirated in abdominal tap and general surgeon was called in for suspecting it to be a major blood vessels injury. Urgent laparotomy was done and a 1 cm rent was identified in the antero-lateral and postero-medial wall of the inferior vena cava at the level of junction of common iliac vessels (Figure 1). The torn vessel was compressed manually to minimize the blood loss till a vascular surgeon arrived. Hemodynamic status of the patient was maintained by giving massive blood transfusion. The vascular surgeon repaired the antero-lateral rent but the postero-medial defect on the under surface could not be repaired. At this time the vascular surgeon decided to isolate the tear by ligatures and do an end to end anastomosis of common iliac vessels with the gonadal vessels to maintain the circulation (Figure 2). The patient was kept in ICU for observation for next 5 days. Over all she received 20 units of whole blood and 35 units of fresh frozen plasma. Abdominal drain was removed on day 5 when the 24-hour collection was less than 100ml. The ultrasound of the abdomen on the subsequent day revealed minimal collection. The patient was maintaining her vitals well however she developed swelling in her left calf and thigh 10 days post surgery for which she was given compression stockings. The patient was discharged in a stable condition from hospital 14 days post surgery after all stitch removal. Her radicular pain had disappeared post surgery. 

## 3. Discussion

Injuries to the surrounding soft tissue vital structures have been occurring ever since surgeons started performing lumbar disc surgeries. The most prominent and feared complication has been the vascular injuries because of their catastrophic effect. Mortality as high as 100%, due to hypovolemic shock has been reported in literature (2). Most of the mortalities occur on the operating table itself. Less serious vascular complications can manifest as arteriovenous fistula or pseudoaneurysm, which may go unnoticed initially and the patient may present even months and years after the event (3). Arterial injuries are more common than venous injuries and are easier torepair as the vessel wall is thick. As the aorta bifurcates at the level of L4-L5 intervertebral space, most of the injuries are encountered in the right or left common iliac arteries (4). Since the blood loss is directly related to the caliber of the injured vessel, injury to iliac vessels have a better prognosis than injury to larger vessels like aorta and vena cava. Most of the vascular injuries occur for discectomies at L4-5 (5-7). The classical diagnostic signs of a vascular injury are hypotension with tachycardia signifying hypovolemic shock. Unfortunately in a young and healthy individual these may not be evident until 30-40% blood has already been lost (8). Excessive bleeding into the surgical field, which is a quicker diagnostic sign, may not occur in upto 40% of the cases, as happened in our case (9). Most of the injuries result from damage caused by the disc biting forceps. An aggressive technique and a haste to complete the case may be responsible for the damage (7). An inaccurate depth perception under a microscope can be another causative factor. But as with most accidents in true sense, likely cause may not be evident all the time. Every surgeon must analyze the situation himself and learn from his and others' mistakes too.

Majority of such vascular injuries are dealt with primary repair of the defect by a vascular surgeon; however in our case the rent was big and placed on the undersurface making it very difficult for the vascular surgeon to approach or repair it primarily. After repeated failed attempts of primary repair, the vascular surgeon decided to the anastomoses as described to maintain the blood circulation and control the bleeding. It may be concluded from this article that:

1. Iatrogenic major blood vessels injuries during posterior spine surgery though rare do happen even in the best of hands.

2. A sudden fall in blood pressure even in absence of blood in the operative field should raise a suspicion of a major blood vessel injury. Active bleeding in the surgical field may only be seen in half of these cases.

3. Do not assume the annulus to afford significant resistance to your instruments while performing posterior discectomy.

4. Always do routine blood grouping and saving for all your spine patients.

5. The significance of a good team work among spine surgeons, vascular surgeons, anesthetists, blood bank technicians and other OT staff cannot be under estimated in such hard times and nothing can be more precious than a patient’s life.
